# Correlations Between Morpho-structural Properties of the Brain and Cognitive and Motor Deficits in Individuals with Traumatic Brain Injury

**DOI:** 10.1089/neur.2024.0091

**Published:** 2025-01-24

**Authors:** Alaleh Alivar, Soha Saleh, Michael Glassen, Easter S. Suviseshamuthu, Vikram Shenoy Handiru, Didier Allexandre, Guang H. Yue

**Affiliations:** ^1^ Department of Radiology, Center for Advanced Imaging Innovation and Research (CAI2R), New York University School of Medicine, New York, New York, USA.; ^2^ Department of Radiology, Bernard and Irene Schwartz Center for Biomedical Imaging, New York University School of Medicine, New York, New York, USA.; ^3^ Department of Rehabilitation and Movement Sciences, School of Health Professions, Rutgers University, Newark, New Jersey, USA.; ^4^ Department of Neurology, Robert Wood Johnson Medical School (RWJMS), Rutgers University, Newark, New Jersey, USA.; ^5^ Center for Mobility and Rehabilitation Engineering Research, Kessler Foundation, West Orange, New Jersey, USA.; ^6^ Department of Physical Medicine and Rehabilitation, Rutgers University New Jersey Medical School, Newark, New Jersey, USA.

**Keywords:** balance impairments, cognitive deficits, fractional anisotropy, fractal dimension, partial least squares correlation, traumatic brain injury

## Abstract

Traumatic brain injury (TBI) results in changes in brain networks followed by long-lasting behavioral and social impairments. This study explores the relationship between neurobehavioral as well as physical function deficits and structural changes in brain white matter (WM) and gray matter (GM) in individuals with TBI by evaluating morphometric magnetic resonance imaging (MRI) and diffusion tensor imaging (DTI) data. The structural MRI-based fractal analysis has emerged as a promising new approach to measure the morphology of the WM and GM. While DTI metrics reflect the microstructural properties of WM, the fractal dimension (FD) is regarded as a measure of morphometric complexity of the system, thus providing complementary information on the brain structure. This study included 10 individuals having moderate-to-severe TBI with balance/postural control deficits and 8 healthy controls. The network-based GM and WM morphologies were measured using FD and structural connectivity metrics, and fractional anisotropy (FA) was assessed using DTI in major WM tracts. The associations between brain structural (FA and FD) measures and a number of neuropsychological assessment and sensorimotor function outcomes were evaluated using partial least square correlation analysis. Our findings showed that the complexity in GM of default mode network, salience network, sensorimotor network, and frontoparietal network is positively correlated with the performance in cognitive and balance outcomes in patients with TBI. On the contrary, in DTI connectivity measures, only few regions including corona radiata, inferior longitudinal fasciculus, and middle cerebellar peduncle were strongly correlated with the behavioral outcomes in the TBI group. Our study suggests that the brain structure complexity measured by FD is a promising and complementary approach to DTI for potentially serving as a biomarker of cognitive and sensorimotor functions in TBI population.

## Introduction

Each year, about 1.7 million people suffer from traumatic brain injury (TBI), and ∼5.3 million individuals currently live with disabilities caused by TBI in the United States.^1–3^ In addition to being a major cause of death and disability in the United States.^4,5^ TBI disrupts the structure and function of the brain leading to both cognitive and sensorimotor impairments, such as memory loss, decreased cognitive processing speed, poor attention, and motor function impairments.^6–9^ If this causal relationship could be thoroughly investigated, the outcome may help develop more effective interventions to restore cognitive and sensorimotor functions in TBI survivors. After TBI, all patients are required to be assessed by a professional with a prognostic toolset to evaluate motor and sensory skills immediately following TBI and over the course of recovery. Diagnostic and prognostic inaccuracy could negatively impact patient’s recovery since it may affect their chance to receive proper rehabilitation treatments based on their specific neurological and physical deficits.

The most consistent characteristic of brain tissue injury post-TBI is diffuse axonal injury (DAI), which is difficult to measure by clinical tools.^10^ DAI measurement can be an important predictor of functional outcome and plays a fundamental role in TBI pathophysiology.^11^ At the time of injury, axons and membranes are damaged by initial shearing forces, and this disrupts axonal transport followed by brain network dysfunction. These injuries are seldom visible on computed tomography and conventional magnetic resonance imaging (MRI) scans. Several neuroimaging modalities have been used to help enhance the prognosis of recovery post-TBI.^12,13^ Advanced MRI methods now allow better detection of DAI, and it has been utilized in identifying brain tissue abnormalities, especially white matter (WM) changes following TBI.^14,15^ Researchers have been exploring novel neuroimaging biomarkers for more accurate detection of brain tissue and structural alterations for diagnosis and prognosis purposes.^16–18^ Multiple imaging modalities would help identify changes in brain structures post-TBI and the effect of it on cognition and physical behaviors. Diffusion tensor imaging (DTI) is an established imaging modality for identifying WM diffuse injuries by measuring fractional anisotropy (FA) as a proxy for WM integrity.^19^ Although there are studies that explored the associations between DTI measures and outcomes of cognitive and balance assessments, only a few have investigated these associations in TBI populations.^20–23^ According to them, the lower the WM anisotrophy in some ROIs (such as the cerebellum, corticospinal tract (CST), and posterior thalamic radiation) is, the poorer the postural control performances are.^20–22^ Additionally, few studies have compared the WM integrity and neuropsychological (NP) behaviors in patients with TBI using either the anisotropy parameters or tract-based statistical analysis.^23,24^ It has been reported that the information processing speed and executive abilities were strongly associated with the WM degradation in some WM ROIs/pathways, namely, corpus callosum, cingulum, superior longitudinal fasciculus, and centrum semiovale. Even though earlier studies highlighted the effect of damages in the brain structure following TBI on cognitive and sensorimotor function impairments, the relationship between WM structural integrity and cognitive or sensorimotor function impairments is too complex to be captured by only DTI structural connectivity measures in the acute phase of injury and in mild TBI. This limitation makes DTI a relatively insensitive biomarker of TBI, thereby limiting its diagnostic and prognostic values.^25,26^ To date, the WM system integrity using DTI in TBI and its correlation with NP and sensorimotor function outcomes has only been studied without jointly investigating the gray matter (GM) and WM structural complexity alterations caused by TBI. Therefore, we suppose that evaluating both the WM and GM structural changes might capture more injury-related features in the brain structure following TBI.

A novel approach to quantitatively represent the brain structure or morphology (e.g., GM and WM structures) is fractal dimension (FD), which is defined as the complexity of any irregular object that has self-similarity.^27,28^ Thus, FD is a unit-less and geometric shape feature that describes the structural details of an object as a single quantitative index of morphometric variability and complexity.^29,30^ The FD in the context of brain structures was first studied by Hofman,^31^ and later others used this feature to explore brain shape and structural complexity based on T1- and T2-weighted magnetic resonance (MR) images.^32–43^ The FD has been used in different studies to estimate both WM and GM complexity changes due to different neurological diseases such as small vessel disease,^43^ aging,^44^ Alzheimer,^37,45^ multiple sclerosis,^33,46^ stroke,^42^ schizophrenia and bipolar disorders,^47^ and TBI.^48^ Interestingly, Rajagopalan et al.^48^ analyzed both FD and DTI data to characterize morphometric complexity of brain WM along with GM volume and cortical thickness in TBI. They compared the FD estimated for WM (segmented and skeletonized from MRI) from each hemisphere (left and right) and the whole brain between TBI and healthy participants and reported a significant reduction in the FD value in TBI, thereby suggesting the use of FD as a potential biomarker of TBI. Motivated by this finding, we set out to investigate the ROI-based FD analysis in both cortical and subcortical areas, as a complementary method to DTI, and explore the relationship between the FD estimate of brain morphology and cognitive performance and balance/postural control dysfunction in patients with TBI.

None of the previous studies have explored the associations between ROI-based brain morphostructural properties and cognitive or sensorimotor function outcomes in TBI populations. To bridge this knowledge gap, we proposed to examine whether damages to specific brain networks/regions are associated with deterioration in cognitive and balance functions in TBI. Therefore, the aim of this study was to assess whether the FD and DTI features of WM and GM in different brain ROIs would correlate with cognitive and balance performance in individuals with TBI. The FD analysis was performed for the following major resting state brain networks: default mode network (DMN), sensorimotor network (SMN), frontoparietal network (FPN), and salience network (SN). For the DTI analysis, we studied the FA parameter within the ROIs that enclose major WM tracts between the two hemispheres (corpus callosum), between the anterior and posterior brain regions (superior and inferior longitudinal fasciculus), and between motor cortices and the spinal cord (CSTs). To examine how well the cognitive and balance deficit measures are correlated with the FD and DTI data, we performed the partial least square correlation (PLSC) analysis.

## Materials and Methods

### Participants

The study included 10 individuals with chronic TBI and 8 age-, height-, and weight-matched healthy controls. Participants were recruited through our in-house patient information management system and Kessler Institute for Rehabilitation. A summary of the demographics is presented in Table 1. Inclusion criteria for participants were as follows: (1) age between 18 and 65 years (children younger than 18 and individuals older than 65 years were excluded to avoid possible effects of brain development in children and aging-related nervous system degeneration on the outcome measures), (2) diagnosed with TBI at least 6 months before participation, (3) medically stable in the past 3 months, and (4) being able to stand unsupported for 5 min. Exclusion criteria were as follows: (1) history of lower limb injury in the past 90 days, (2) history of medication that could affect the balance/muscle coordination and cognitive function, (3) history of any additional orthopedic, neuromuscular, or neurological conditions that could affect balance/cognitive functions, (4) history of a penetrating TBI, (5) history of previously diagnosed balance and cognitive function impairments (prior to TBI), and (6) having any metal implants or MRI contraindications. The study was reviewed and approved by the Kessler Foundation Institutional Review Board. All participants signed informed consents before participating in the study.

**Table 1.** Demographic and Clinical Characteristics of the Sample

	HC (*n* = 8)	TBI (*n* = 10)
Gender (M/F)	5/3	8/2
Age (mean ± std)	49 ± 10.6	47.7 ± 13.1
Height (cm) (mean ± std)	173.1 ± 7.9	180.6 ± 6.4
Weight (lbs.) (mean ± std)	161.9 ± 26.1	189.3 ± 38.2
BBS (mean ± std)	56 ± 0	50.2 ± 5.6
COP (mean ± std)	8.7 ± 2.6	10.6 ± 3.9
TMT (mean ± std)	41.8 ± 21.1	41.6 ± 6.2
SDMT (mean ± std)	36.4 ± 7.8	32.7 ± 5.9
SCWT (mean ± std)	38.9 ± 8.1	53.1 ± 10.6

BBS, Berg Balance Scale; COP, center of pressure displacement; SCWT, Stroop Color and Word Test; SDMT, Symbol Digit Modalities Test; TMT, Trail Making Test.

### MRI data acquisition

High-resolution T1-weighted images with an MPRAGE sequence were acquired using the Siemens Skyra 3T Scanner (Erlangen, Germany) in Rocco Ortenzio NeuroImaging Center at Kessler Foundation. The following settings were used in the whole-brain volumetric acquisition with 1 mm isotropic voxel resolution: TE = 3 ms, TR = 2300 ms, 176 1-mm thick slices, 256 × 256 mm FOV, and 256 × 256 matrix.

A diffusion-weighted (DW) dataset was collected with 64 noncollinear DW gradients with b = 1100 s/mm^2^ and 8 b = 0 images, matrix size = 128 × 128, FOV = 256 × 256 mm^2^, TE = 75 ms, TR = 9000 ms, isotropic voxel dimensions = 2 mm, and 66 slices. The gradient-echo field map images were acquired to correct for geometrical distortion caused by susceptibility artifacts.

### DTI fractional anisotropy

The image processing was performed using the FSL 6.0 toolbox that includes the following steps: (1) skull stripping using the Brain Extraction Tool, (2) eddy current correction, and (3) DTIFIT diffusion tensors using FDT (FMRIB’s Diffusion Toolbox, http://www.fmrib.ox.ac.uk/fsl, Oxford, UK). The generated results were parametric maps of DTI measure including FA. Next, the ROI-based FA values for each subject were obtained by averaging the corresponding FA values of the voxels in the respective ROI mask, using FSL math.^49^ The explored ROIs were extracted from the JHU DTI-based WM atlas^50–52^ that included the following: bilateral CST, bilateral super longitudinal fasciculus, body of corpus callosum, the genu of corpus callosum, splenium of corpus callosum, caudate, bilateral cingulum, bilateral inferior occipital fasciculus (inferiorOccF), bilateral corona radiata, bilateral inferior longitudinal fasciculus (inferiorLongF), and middle cerebellar peduncle (MCP).

### Fractal analysis

Cortical reconstruction and volumetric segmentation were performed using the FreeSurfer image analysis suite, which is documented and freely available for download from the following link: http://surfer.nmr.mgh.harvard.edu/. The technical details of these procedures are described in other publications.^53–55^ This pipeline was applied to subjects’ T1 scans (i.e., recon-all command with all option). No manual edits were made. The networks chosen for analysis are DMN, dorsal attention network, FPN, SMN, and SN. Both GM and WM in these networks were considered for FD estimation. Subsequently, the reconstructed segmentations were included in FD calculation for each region and each subject. We performed a three-dimensional FD analysis using box-counting algorithm,^56^ which is a fairly simple and reliable method to estimate the FD of an object with or without self-similarity such as the human brain. The FD estimation via box-counting procedure is briefly described below. To begin with, a series of cubes with the side length *r* are overlaid to enclose a brain ROI, and the number of cubes required, *N*(*r*), is recorded as a function of *r*. Next, this counting procedure is iteratively applied with different values of *r* and the respective *N* values are noted. Finally, the FD is estimated as the absolute value of the slope of the linear regression model for the log-transformed relation between *N* and *r*, as given in Eq. (1)^56^ with *K* and *FD* being the prefactor and FD, respectively.

(1) $$N\left(r\right)=Kr^{-FD}\Rightarrow \;\ln\;N=FD\;\ln(1/r)+\ln\left(K\right)$$

**FIG. 1.**
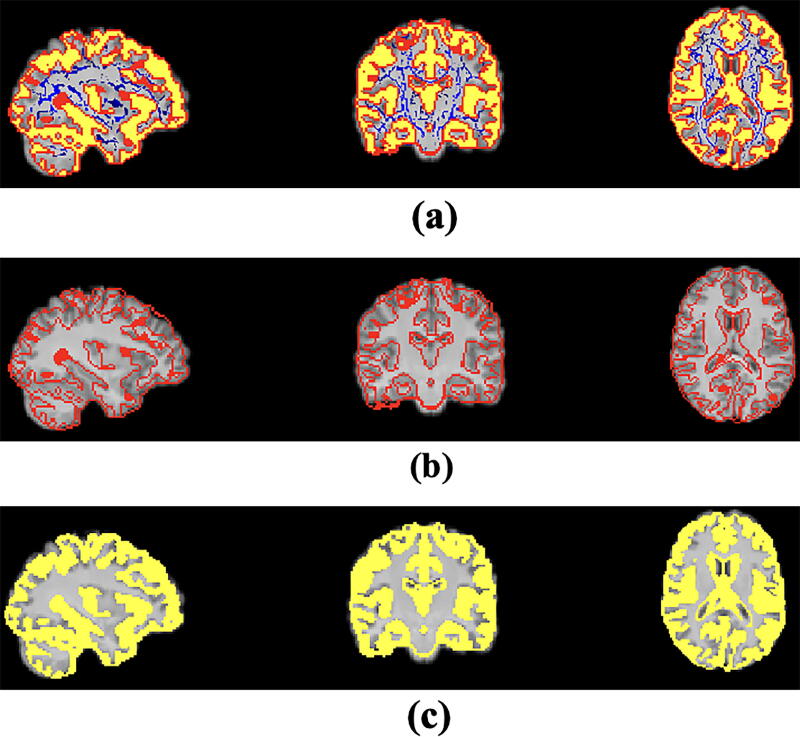
**(a)** First row is showing all three features of GM general structure, GM surface, and WM skeleton overlapped on skull-stripped T1-weighted image. Individually, **(b)** the second row is showing the whole brain GM surface (red), and **(c)** the third row is the general structure (yellow). GM, gray matter; WM, white matter.

Using this approach, we estimated FD of three structural features—surface, skeleton, and general structure—for each region. The *skeleton FD* was estimated by counting the cubes needed to cover the WM skeleton in each region, which represents the interior structure complexity of the region. The *surface FD* was estimated by counting the cubes covering the surface of the structure, which is the external surface of the GM (pial surface) or WM/GM interface. The *general structure FD* was estimated by counting the cubes needed to enclose the entire volume of GM in the region (including the surface). An example of GM surface, general structure of whole brain GM, and skeleton of WM maps are shown in Figure 1.

### NP evaluation

Three NP assessments that are sensitive to attention and executive functions in patients with TBI were administrated by a trained research assistant in the Center for Mobility and Rehabilitation Engineering Research at Kessler Foundation. The *Trail Making Test* (TMT), a visual attention and task switching test, was used to measure executive functions.^57–59^ The *Symbol Digit Modalities Test* (SDMT), a sustained attention task, was used to measure information processing speed.^60^ The *Stroop Color and Word Test* (SCWT) assessed the ability to inhibit cognitive interference.^61,62^ The summary of average scores for these three tests for each group is presented in Table 1.

### Balance assessments

Assessments of balance function were performed using *Berg Balance Scale* (BBS), a 14-items scale for evaluation of balance function,^63^ and by measuring *center of pressure (COP) displacement* during a perturbation test on a computerized dynamic posturography (CDP) platform.

#### Setup for the COP displacement measurement

The CDP platform (NeuroCom Balance Master, NeuroCom Intl, Clackamas, OR) was used to evaluate the effect of unpredictable postural perturbation on an individual’s COP. The setup included participants standing on the balance platform and being exposed to five blocks of random unpredictable perturbations in two directions, anterior (forward) and posterior (backward). The perturbation consisted of a sinusoidal wave shape movements of the platform with 0.5 Hz frequency (two cycles for a total of 4 sec duration). Participants were tested with a low (0.5 cm) and high (2 cm) amplitude perturbations with an inter-trial separation of 4–8 sec. Thus, participants were tested in a setting of 2 × 2 combinations of perturbations (anterior and posterior) and amplitudes (low and high). The COP time series was estimated using the ground reaction force data of the balance platform. The time series was preprocessed and averaged across trials and conditions for each subject. The preprocessing included epoching the events, low-pass filtering the raw signal (10 Hz cutoff), and detrending the signal. The COP displacement was computed as the cumulative distance traveled by the trial averaged COP in the anterior/posterior direction for the first 2 sec of the balance perturbation. In this article, the analysis is limited to the high-amplitude perturbation in the posterior direction, which led to greater instability across all subjects.

### Statistical analysis

The statistical analysis was performed using the MATLAB R2019a statistics and machine learning toolbox. A two-tailed Student’s *t* test was used for between-group comparisons of demographic and cognitive and balance data. The uncorrected significance level was set to *p* value ≤0.05 for these analyses. The multivariate relationship between behavioural deficits and structural imaging measures (FD and DTI) was examined using the PLSC analysis.

#### Behavioral partial least square correlation

Behavioral PLSC allows to identify the structural imaging measures that would best correlate with behavioral variables without having to perform multiple correlation tests and increase the risk of Type I error.^64^ The output is a short list of imaging measures that correlate with behavioral measures and the direction of relationship (positive or negative). In this study, PLSC was performed between the brain imaging measures (DTI-based FA and FD) and the behavioral measures—combination of balance assessment measures (namely, BBS and COP) and cognitive assessment measures (namely, TMT, SDMT, and SCWT).

For each subject, the DTI measures from 18 ROIs and FD measures from 16 ROIs are concatenated to form the “brain imaging data” (size: 1 × 34). Similarly, the two balance outcome measures (BBS and COP) and three cognitive measures (TMS, SDMT, and SCWT) are concatenated to form the “behavioral” measures (size: 1 × 5). Thus, the brain imaging data aggregated from all subjects across TBI and HC groups form the matrix “*X*” of size 18 × 34, and the behavioral data forms the matrix “*Y*” of size 18 × 5. The cross-block covariance matrix *R* resulting from $Y^{T}X$ is then subjected to the singular value decomposition $\left(R=U\Delta^{T}V\right)$ within the framework of behavioral PLSC, where *U* is the left singular vector, Δ is the matrix of singular values, and V is the right singular vector. To implement PLSC, we used the myPLS toolbox,^65^ which is based on the PLSC toolbox developed by McIntosh et al.,^66^ and the analysis was similar to that reported in previous studies.^65,67^ A total of 1000 permutations of the PLSC analysis was performed in the toolbox to confirm the statistical significance of latent components (LCs) resulting from the singular value decomposition of cross-block covariance matrix. In this multivariate framework, each LC is characterized by a distinct pattern of brain imaging features (called “imaging saliences”) and behavioral features (called “behavioral saliences”) related to the right and left singular vectors (*U* and *V*), respectively. We can obtain a composite “imaging score” and “behavioral score” by linearly projecting the brain imaging data (*X*) and behavioral data (*Y*) onto the respective saliences (right and left singular vectors). Furthermore, we can obtain the imaging (or behavioral) “loadings” by estimating the correlation between the imaging (or behavioral) salience and the imaging (or behavioral) measures. The design saliences are reported in the results to summarize the relationship between the DTI and FD measures, on one hand, and each of the three cognitive assessment scores and two balance assessment scores, on the other hand.

## Results

The following sections report the results of the group comparisons and significant correlations using FD and FA measures in predefined networks and regions.

### NP and postural/balance performance

**FIG. 2.**
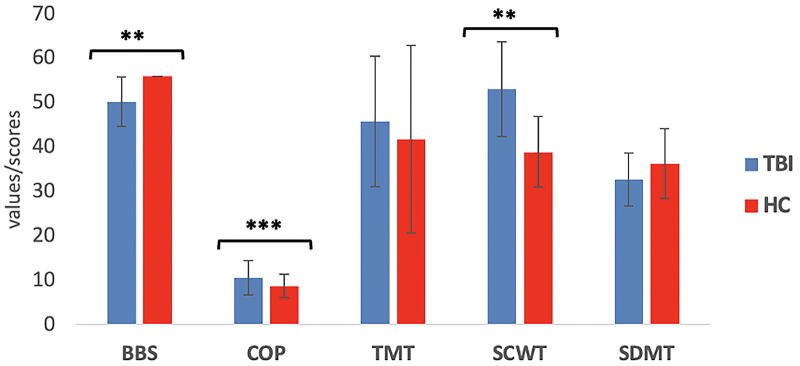
Group comparison of balance and neuropsychological outcomes in patients with TBI and healthy controls; mean and standard deviation for each domain are presented (*p value < 0.05, **p value < 0.01, and ***p value < 0.001). TBI, traumatic brain injury.

Participants with TBI displayed significantly poorer performance (*p* = 0.009) in BBS and COP (*p* = 1.6e^−6^) balance test outcomes than the HC group (Fig. 2). Regarding the NP data, we found significantly higher SCWT (worse performance) in TBI than the HC group (*p* = 0.005, *η* = 1.5). No significant group differences were found for TMT (*p* = 0.65, *η* = 0.22) and SDMT (*p* = 0.3, *η* = 0.52) measures across groups.

### GM FD and DTI correlation with behavioral outcomes

**FIG. 3.**
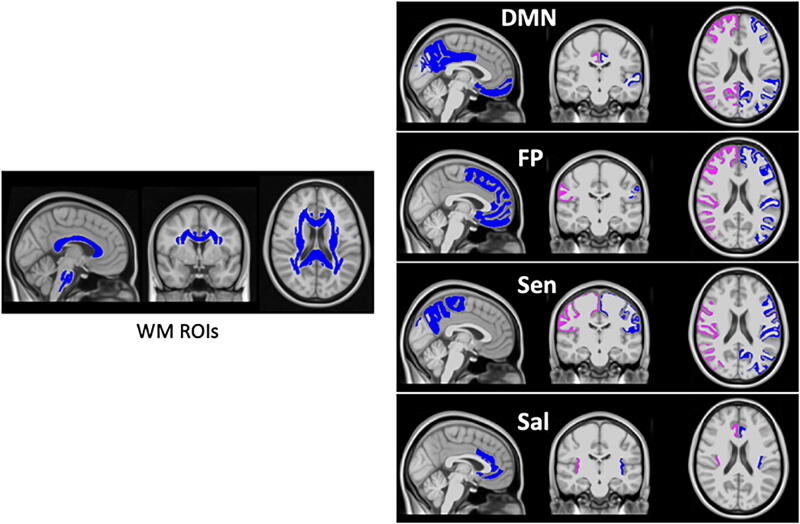
Brain white matter ROIs from JHU-ICBM atlas and networks including DMN (default mode network), FP (frontoparietal), Sen (sensorimotor), and Sal (salience) overlapped on T1 MNI152. Blue and pink regions are right and left hemisphere, respectively, for the right panel.

**FIG. 4.**
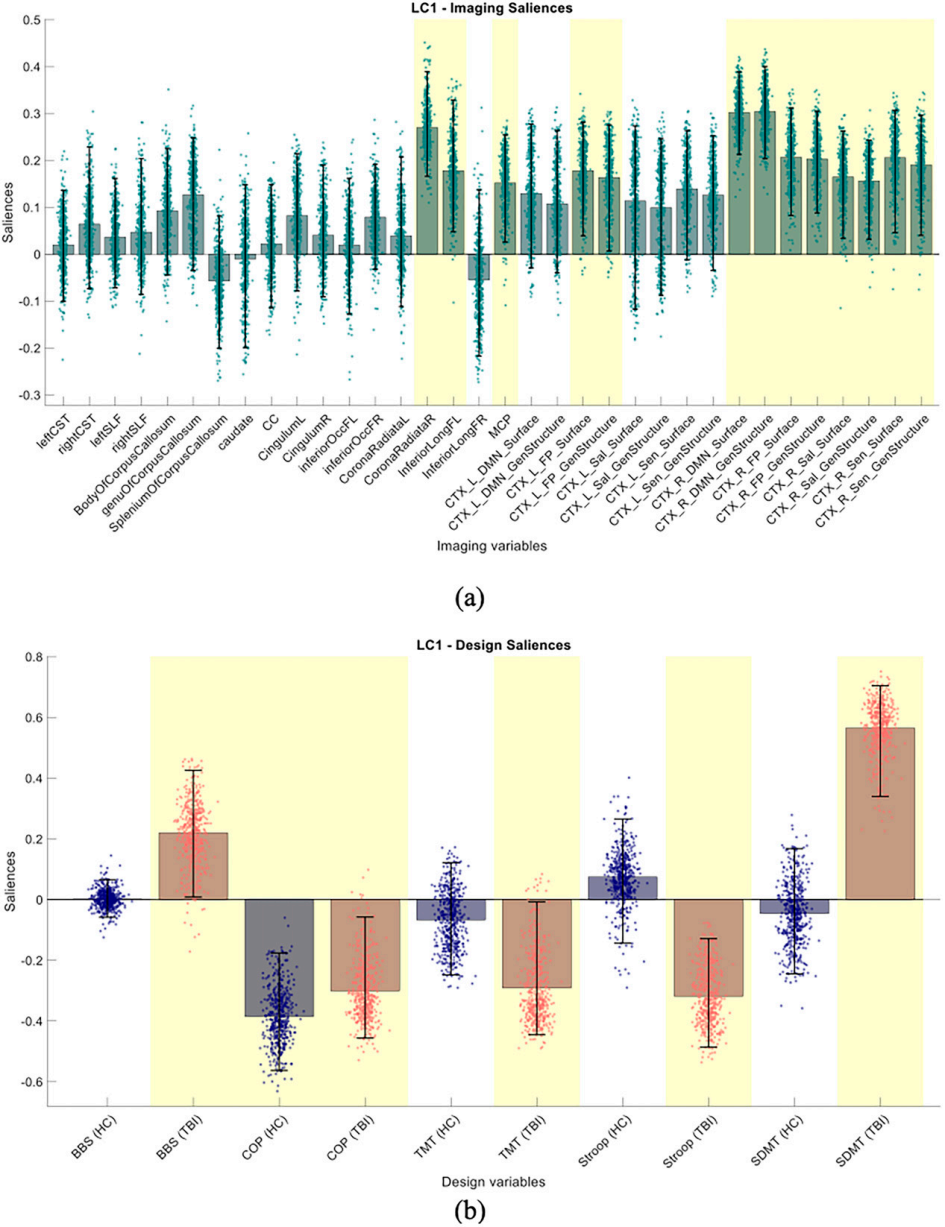
PLS correlation analysis of brain measures (FD and FA measures) with balance and cognitive measures resulted in one significant LC (*p* value < 0.001). **(a)** Brain saliences of each of the brain measures (FA and FD). Measures highlighted in yellow are significantly associated with design saliences in **(b)** after bootstrapping. **(b)** Design saliences showing significant association (correlation) between cognitive measures (TMT, SDMT, and Stroop) and brain measures only within the TBI group, association between brain measures and BBS only in the TBI group, and association between brain measures and COP measure of balance in both groups. COP, center of pressure; FA, fractional anisotropy; FD, fractal dimension; LC, latent components; PLS, partial least squares; SDMT, symbol digit modalities test; TBI, traumatic brain injury; TMT, Trail Making Test.

**FIG. 5.**
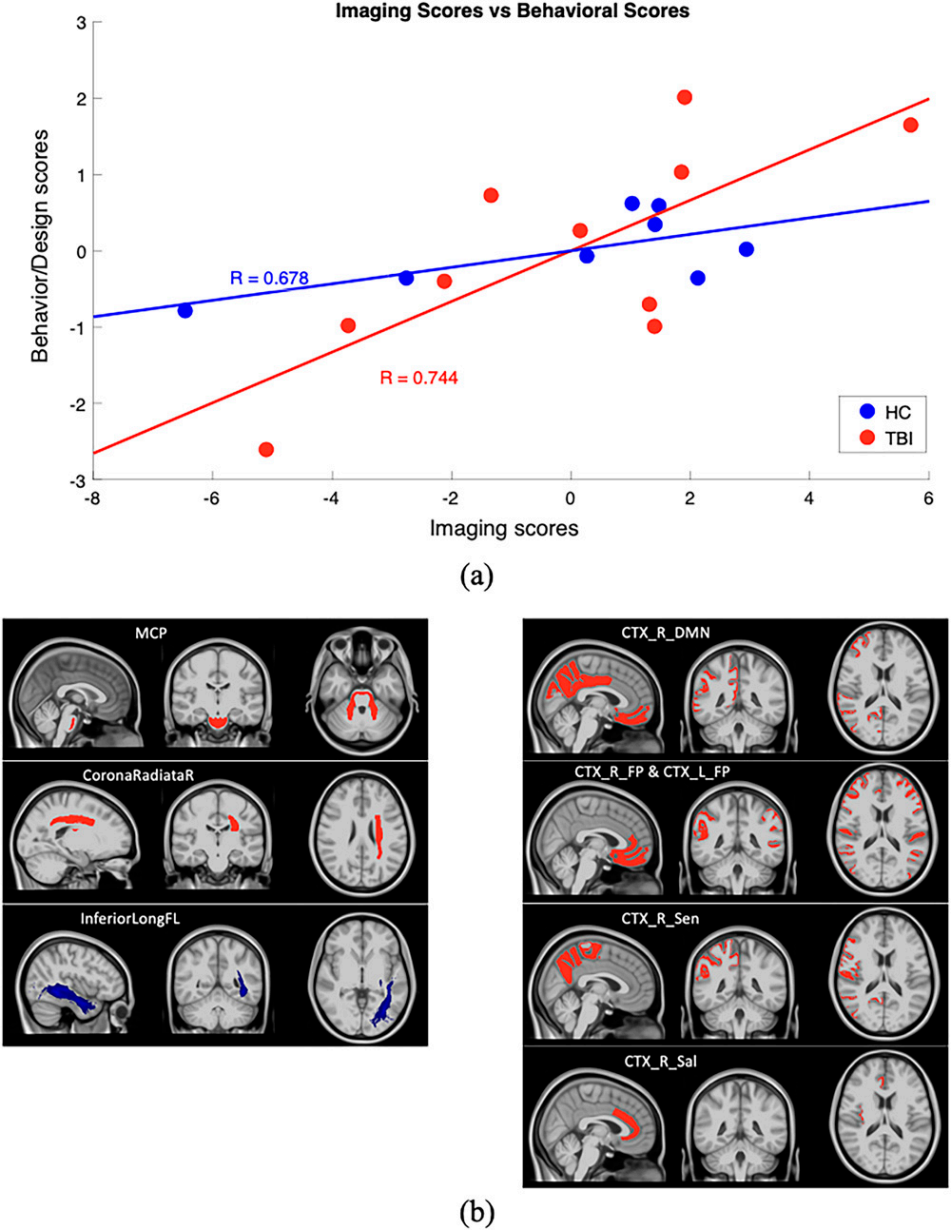
**(a)** Correlation between brain (imaging) scores and design measures (behavioral scores). The direction of correlation suggesting higher FD and FA values in subjects with better behavioral scores. **(b)** The brain ROIs and networks showing significant associations with design saliences in Figure 4(a) in which red is demonstrating HC < TBI and blue is HC > TBI, overlapped on T1 MNI152. FA, fractional anisotropy; FD, fractal dimension; TBI, traumatic brain injury.

Behavioral PLSC analysis was conducted to examine the relationships between brain imaging features (comprising FD and DTI measures from different regions of interest) (see Fig. 3) and the behavioral measures (comprising NP and balance outcome measures) as explained before. Upon nonparametric permutation testing, we obtained one significant LC (*p* = 0.03, suggesting the empirical singular value is in the top 3% of the histogram of singular values obtained using permutation testing). This significant LC explained 50.7% of the covariance. The results of the significant LC are presented in Figures 4 and 5, illustrating the important brain imaging saliences and behavioral saliences, respectively.

As shown in Figure 4a, in the TBI group, mostly the GM complexity of FP, DMN, SN, and SMN is correlated with the behavioral outcomes; however, only a few DTI connectivities in corona radiata, inferior longitudinal fasciculus, and MCP show association with design saliences (regions are shown in Fig. 5b). These features are shaded in yellow to indicate that they are deemed “stable.” The stability of each feature has been determined by checking if its value divided by its standard error deduced via bootstrap resampling exceeds a threshold of 2.^64^ In simple words, the exclusion of “stable” features may change the results completely, whereas excluding the nonstable features would not affect the PLSC findings. Also, in the TBI group, the brain structural complexity is correlated with the behavioral assessments of balance and cognition (Fig. 4b). In other words, the brain complexity in the TBI group is positively correlated with the SDMT and BBS measures and negatively correlated with the SCWT, TMT, and COP values. The COP was considered “stable” in both groups meaning that in both groups, the FD of FP, DMN, SN, and SMN in GM was negatively correlated with COP (see Figs. 4a and 5b). Interestingly, the TMT and SDMT measures were not statistically different between groups (Fig. 2), but they show different relationships with brain connectivity measures (see Fig. 4b). Also, correlation between brain (imaging) scores and design measures (behavioral scores) is shown in Figure 5a in which the direction of these correlations in both the HC and TBI groups suggest higher FD and FA values with better behavioral scores.

## Discussion

Our work showed that FD can be informative in evaluating the relationship between brain structural changes and cognitive and motor impairments in individual patients with TBI. FD was not suggested as an alternative method to DTI but rather a different methodology that provides complementary information suggesting how complex a structure is. In the past decade, there has been an increase in the number of studies using fractal analysis to better address the structural complexity changes across different neurological populations.^10,31,68–75^ However, to our knowledge, none has performed such analysis in TBI population focusing on the correlation between ROI-based FD and cognitive and balance performance outcomes. One of the main advantages of using ROI or network-based brain analysis is to provide important information on the underpinnings of plasticity, adaptation, and response to brain injury and rehabilitation intervention, and these findings are expected to contribute to the development of advanced rehabilitation interventions.

Our results showed that the motor and cognitive functions in the studied participants are associated with FD measures of GM for a number of networks; however, this correlation has not been observed in most DTI connectivity measures. These findings suggest that the FD-based GM complexity measurement of one or more brain regions can give additional information about the brain structure integrity, which could help predict motor and cognitive functions in individuals with TBI.

Furthermore, TMT and SDMT measures did not show any difference between HC and TBI, but they showed a difference in correlation direction with brain measures suggesting that FD measures can be more sensitive in identifying abnormalities in brain measures even when the behavioral abnormalities are mild and the outcome measures do not show a significant difference between healthy and patient groups.

### Cognitive outcomes

The results provided evidence of significant correlation between cognitive outcomes of executive function and FD measures for different brain networks. The observed correlation between cognitive processing speed and GM FD of DMN can be explained by the fact that DMN consists of regions with dense WM connections^76^ and includes regions in the frontal and parietal cortices that are involved in modulating cognitive and attention tasks. Thus, the results of relationship between complexity of DMN GM structure and SDMT could be related to functional activity of the brain structure to control cognition and attention^77^ and suggest that the performance in information processing (lower SDMT score) is positively correlated with the structural complexity in DMN, which could be an indication of the GM degeneration following TBI.

DTI FA in corona radiata, inferior longitudinal fasciculus, and MCP showed significant relationships with cognitive outcomes. Since WM disruption as a result of DAI can be distinguishable from tract disconnections, we also explored the relationships between FA of major WM tracts and NP outcomes. Based on the FA results, we showed that the damages in WM connectivity of few regions correlated significantly with poorer cognitive and balance performance in individuals with TBI. This finding in fact is in line with previous researches that showed that WM structural changes are highly correlated with and influence the processing speed.^78–80^ In conclusion, FD and DTI measures are both sensitive to different brain structural changes, and therefore, they can explain behavioral measures differently.

### Balance function outcomes

Recent studies reported a relationship between changes in structural connectivity and balance deficits post-brain injury using DTI and tractography measures.^21,22^ Previously unreported, we observed significant association between balance deficits and brain structural complexity in patients with TBI using region-based morphological measures of fractal analysis. Worse performance in postural control, indicated by larger COP displacements, was observed in participants with lower FD or complexity level in the GM of SMN, FPN, and DMN. This result is not surprising knowing that these networks are important for balance function. SMN is involved in controlling and execution of motor tasks, while FPN is more involved in motor planning and attention to external and internal environment.^81,82^ Relationship with DMN is also not surprising since the DMN is upregulated or downregulated during different tasks, playing a major role in modulating dual tasks^83^ such as posture behavior. Most importantly, this study suggests that FD could serve as a potential marker of balance/postural deficits post-TBI and describe the brain morphological and structural internal shape changes, while few correlations were found between the DTI measure of WM connectivity and balance outcomes.

### Limitations and future works

Besides the significant findings of the present study, there were a number of limitations. First, we had a small number of subjects; therefore, additional studies with more participants would be needed to corroborate our results. We used PLSC to address this issue and allow performing multivariate analysis. Future works with a larger sample size (to avoid overfitting) may explore both diffusivity and FD measures in one regression model to predict the outcomes and evaluate the importance of each feature in predicting the behavioral outcome post-injury. Also, it is important to study longitudinal data to understand how structural abnormalities (both cortical and subcortical) vary with time or rehabilitation intervention following the injury.

## Conclusions

The present study provides cortical and subcortical ROI-based morphological and structural information of the brain and the corresponding associations with cognitive and balance performance outcomes post-TBI. It provides evidence that FD can be particularly useful to explore the morphostructural properties of the brain in TBI and potentially serve as a marker of balance dysfunction in the TBI population. This study shows that DTI measures of WM connectivity and FD measures of cortical structural complexity show different relationship with behavioral measures and can be complementary as biomarkers of behavioral function and recovery post-brain injury. Going forward, we hope that this information and analysis would be adopted in larger studies to examine FD measures as potential biomarkers in clinical settings, since FD is an easy measure to acquire, and it encodes significant information about structural complexity of the brain into one single value.

### Transparency, Rigor, and Reproducibility Summary

This study was not formally registered because it was not a randomized interventional study but an observational comparative study to examine the relationship between neuroimaging and behavioral data. The analysis plan in this study was not formally preregistered, but the team member with primary responsibility for the analysis certifies that the analysis plan was prespecified. A sample size of 60 TBI subjects and 20 HC was planned based on the feasibility of completing the data acquisition analysis given the duration of the study and available resources. A total of 200 participants were approached/screened. Thirty-six participants from whom behavioral assessments were collected and 10 TBI and 8 HC participants from whom all measurements were collected, including neuroimaging. Participants were blinded to the results of the assessments throughout the study, even after primary clinical observations were complete. Data analyses were performed by investigators who were aware of subject group. Data were acquired between November 10, 2016 and November 22, 2019. Data were analyzed using Freesurfer, FD toolbox, and FSL. All datasets were analyzed at the same time. No unexpected events occurred during the study. All software used to perform acquisition and analysis are available online. The hardware used is Siemens MRI scanner and NeuroCom Balance platform. which is available from NeuroCom Intl company. The key inclusion criteria (e.g., primary diagnosis or prognostic factor) are established standards in the field. Statistical analysis and/or review was performed by the lead author Dr. Alivar, the research engineer, Michael Glassen with assistance from Dr. Saleh, Dr. Shenoy Handiru, and Dr. Easter Suviseshamuthu, they have qualifications including many years of experience in data analysis and statistical methods. Methods that do not require correction for multiple comparisons were used, including PLSC. There is no replication or external validation studies being performed or are planned/ongoing at this time to our knowledge. Deidentified data from this study are not available in a public archive. Deidentified data from this study will be made available (as allowable according to institutional IRB standards) by emailing the corresponding author Dr. Guang Yue within 5 years of publication of this article. Similarly, the analytic code used to conduct the analyses presented in this study is not available in a public repository. They may be available by emailing the corresponding author or the lead author within 2 years of publication of this article. The authors agree to publish the article using the Mary Ann Liebert Inc. “Open Access” option under appropriate license.
